# Human Norovirus Histo-Blood Group Antigen (HBGA) Binding Sites Mediate the Virus Specific Interactions with Lettuce Carbohydrates

**DOI:** 10.3390/v11090833

**Published:** 2019-09-08

**Authors:** Malak A. Esseili, Xiang Gao, Patricia Boley, Yixuan Hou, Linda J. Saif, Paul Brewer-Jensen, Lisa C. Lindesmith, Ralph S. Baric, Robert L. Atmar, Qiuhong Wang

**Affiliations:** 1 Food Animal Health Research Program, Ohio Agricultural Research and Development Center, Department of Veterinary Preventive Medicine, The Ohio State University, Wooster, OH 44691, USA (X.G.) (P.B.) (Y.H.) (L.J.S.); 2 Currently at Center for Food Safety, Department of Food Science and Technology, University of Georgia, Griffin, GA 30223, USA; 3 Department of Epidemiology, University of North Carolina, Chapel Hill, NC 27599-7435, USA (P.B.-J.) (L.C.L.) (R.S.B.); 4 Department of Molecular Virology and Microbiology and Department of Medicine, Baylor College of Medicine, Houston, TX 77030, USA

**Keywords:** Foodborne viruses, Human norovirus, Lettuce, Histo-blood group antigens, Virus-lettuce interactions, Virus-like particles, mutations, H-like antigens, virus HBGA-binding sites

## Abstract

Lettuce is often implicated in human norovirus (HuNoV) foodborne outbreaks. We identified H-like histo-blood group antigens (HBGAs) on lettuce leaves as specific binding moieties for virus-like particles (VLPs) of HuNoV GII.4/HS194/2009 strain. The objective of this study was to determine whether HuNoV-lettuce binding is mediated through the virus HBGA binding sites (HBS). Toward this objective, VLPs of historical HuNoV GII.4 strains (1987, 1997, 2002, 2004 and 2006) with known natural mutations in their HBS, two newly generated VLP mutants of GII.4/HS194/2009 (D374A and G443A) and a VLP mutant (W375A) of GI.1/Norwalk/1968 along with its wild type VLPs, which displays distinct HBS, were investigated for their binding to lettuce. ELISA revealed that historical GII.4 strains binding to lettuce was dependent on their HBGAs profiles. The VLP mutants D374A and G443A lost binding to HBGAs and displayed no to minimal binding to lettuce, respectively. The VLPs of GI.1/Norwalk/1968 strain bound to lettuce through an H-like HBGA and the binding was inhibited by fucosidase digestion. Mutant W375A which was previously shown not to bind to HBGAs, displayed significantly reduced binding to lettuce. We conclude that the binding of HuNoV GII.4 and GI.1 strains to lettuce is mediated through the virus HBS.

## 1. Introduction

Human norovirus (HuNoV) continues to be the leading cause of foodborne disease outbreaks in the United States (US) [1]. It is estimated that 9.4 million foodborne illnesses occur annually in the US. The majority of these foodborne illnesses (58%) are caused by HuNoV with an estimated 15,000 hospitalizations and 150 deaths annually and over $2 billion in health-care costs [2,3]. Human norovirus is transmitted via the fecal-oral route, including direct person to person, consumption of contaminated food or water or contact with fomites. Infections with HuNoV cause gastrointestinal symptoms (diarrhea, vomiting, stomach pain and nausea) which are usually self-limiting in healthy adults but can be severe and prolonged in young children, elderly and immune-compromised patients [4]. The virus is a small (27–40 nm in diameter), non-enveloped, single stranded RNA virus that belongs to the *Caliciviridae* family [5]. Human norovirus can be classified into at least five genogroups (GI–GV), which are further each subdivided into genotypes [5]. Infections with HuNoV are caused by both GI and GII strains. Among them, the GII.4 HuNoVs are the dominant strains causing the majority (~60–90%) of gastroenteritis outbreaks [6]. Currently, there are HuNoV vaccine candidates undergoing clinical trials [7,8,9]. However, in the absence of approved antivirals or vaccines against HuNoV, the virus continues to exert a significant global burden, estimated at ~$64 billion in direct (healthcare) and indirect (loss of productivity) costs [10].

In the US, analysis of HuNoV foodborne outbreaks that could be attributed to a single food category showed that fruits and vegetables accounted for 51% of the outbreaks [4]. Specifically, lettuce and other leafy greens were most often implicated (30%) in these outbreaks [4]. This is similar to the trend reported from European countries, where HuNoV accounted for 50% of outbreaks in single food commodities of fruits and vegetables and which primarily involved lettuce [11]. Contamination of leafy greens with HuNoVs can occur at any stage along the farm-to-fork chain through a number of sources, including contaminated water used for irrigation or processing, improperly treated sewage sludge used for fertilization and asymptomatically HuNoV-infected food harvesters or food handlers who do not follow proper hygiene practices [12,13]. Because leafy greens are prone to contamination with human pathogens, they are globally recognized to be a high priority in terms of the microbial safety of fresh produce [12]. Our group has shown that many factors enhance HuNoV persistence on the surface of lettuce leaves, including the presence of phytopathogens and physical damage [14,15,16]. The virus was also shown to internalize through the roots of lettuce seedlings and was detected by confocal microscopy inside the leaf mesophyll [17]. In addition, our group has shown that HuNoV GII.4 virus-like particles (VLPs) can bind specifically to lettuce cell wall carbohydrates extracted from leaves [16]. Furthermore, we have shown that lettuce leaves contain H-like histo-blood group antigen (HBGA) but not the A or B antigens [18]. Binding of GII.4 HuNoV VLPs was mediated via a lettuce cell wall hemicellulose and was inhibited with anti-H-HBGA antibody and by digestion with a fucosidase enzyme [18]. Because leafy greens are consumed raw or minimally processed, simple washing may not be enough to remove specifically bound and internalized viruses. Understanding how the virus binds to lettuce would guide efforts to disrupt the virus transmission through this commodity and therefore reduce foodborne illness resulting from consuming lettuce contaminated with noroviruses.

Human HBGAs are complex glycans expressed on the surface of red blood cells and mucosal surfaces of secretor individuals or can be present as free antigens in the biological fluids of secretor individuals such as saliva, milk and intestinal contents [19]. In humans, HuNoV binds to HBGAs which are important for a productive norovirus infection [20]. The majority of GI HuNoVs binds to H- and A-type HBGA and Lewis antigens, whereas GII HuNoVs exhibit a more diverse HBGA binding pattern including both the above and B-type HBGAs [21,22]. The HuNoV genome is ~7–8 kb and organized into three open reading frames (ORF). The ORF1 encodes a large polyprotein which is cleaved into seven non-structural proteins. ORF2 encodes the major structural (or capsid) protein VP1 and ORF3 encodes the minor structural protein VP2. Expression of the VP1 in insect cells generates self-assembled VLPs, which are morphologically and antigenically similar to norovirus virions [21]. Binding of the virus to human HBGAs occurs through the VP1 capsid protein. Specifically, the VP1 is divided into two domains, the shell (S) and the surface protruding domain (P). The P domain is further divided into P1 (residues 222–274 and 418–539), which forms a stalk to project the P domain away from the shell and P2 (residues 275–417) which is the most surface exposed part of the virus [5]. The P2 subdomain contains the HBGA-binding sites (HBS) and some neutralizing antibody epitopes [5]. The VP1 protein sequence from GI and GII HuNoVs can differ by up to 60%, whereas within a genogroup, the genotypes differ by 20–30% [23]. A major difference between the HBS of GI and GII noroviruses is that much of the HBS for GI strains remained intact whereas substantial variation occurred over time within that of GII strains [21]. Crystallography studies with GII.4 showed that HBGA binding involves site 1 (residues 343–345, 374 and 441–444), which mainly interacts with α-fucose of the HBGAs and site 2 (residues 390–395), which stabilizes the interactions along with several other residues proximal to those sites forming a pocket [22,24,25]. Studies with GI.1 HuNoVs revealed that HBGA binding occurs through residues 327, 375, 377, 378 to the β-Gal of H-type HBGA or the GalNAc of A-type HBGA and through residues 329, 342, 344, 346, 375 and 380 to α-fucose of the H- or A-type HBGA [22,26]. For GI.1 HuNoVs, the amino acids at these positions are well conserved but for GII.4 HuNoVs only site 1 residues are conserved. Altered HBGA binding patterns, coupled with extensive antigenic variation in response to herd immunity, have resulted in the emergence of new variants of GII.4 HuNoV every 3–7 years [27]. For example, the first major HuNoV pandemic occurred in 1995 to 1997, which was replaced by subsequent pandemics in 2002, 2004, 2006, 2009 and 2012 [5].Variations in the surface exposed amino acids in the P2 regions correlate with altered HBGA binding capacity and antigenic variation that allows the virus to escape from existing protective immunity [21].

The objective of this study was to determine whether HuNoV binding to lettuce carbohydrates is mediated through the virus HBS. To accomplish this objective, we have assessed the binding of some of the historical GII.4 VLPs (Table 1) to lettuce cell wall carbohydrates to determine whether the changes in the HBGA binding profiles affect their binding to lettuce. Then, using the HuNoV GII.4/HS194/2009 VLP strain that we characterized previously [16,18], we generated two VLP mutants with single amino acids substitutions at residues 374 and 443 located within the HBS. The VLP mutants were assessed for their binding to lettuce. In addition, VLPs for GI.1/Norwalk/1968 (referred to as GI.1), which has distinct HBS and its previously characterized mutant (W375A) that lost HBGA binding [22] were investigated for their potential binding to lettuce.

## 2. Materials and Methods

### 2.1. Production and Purification of HuNoV VLPs

A recombinant baculovirus carrying the VP1 gene (ORF2) of the Hu/NoV/GII.4/HS194/2009/US strain (GenBank accession no. GU325839, genetically closest to GII.4/Minerva/2006 strain)) was generated previously by our laboratory [32]. The VLPs were generated by inoculating insect cells (*Sf9*) with the recombinant baculovirus according to standard protocols described in our previous publications [16,18]. Briefly, after infection of *Sf9* cells with the recombinant baculovirus, cells and media were harvested at 7–10 post-inoculation days (PID). The VLPs were pelleted through sucrose cushions and were further purified by CsCl gradient ultracentrifugation. The protein concentration was quantified using the Bradford method and the morphology of the VLPs was examined by transmission electron microscopy (TEM) after staining with 3% phosphotungstic acid (pH 7.0) [32]. The VLPs of historical GII.4 HuNoVs (1987, 1997, 2002, 2004 and 2006), their detecting primary antibody and HBGA profiles were described previously [21,24,28]. The GI.1 VLP and its mutant W375A, which lost binding to HBGAs, were generated as previously described [22].

### 2.2. Generation of GII.4 Mutant VLP

The two recombinant baculoviruses for the expression of VLP mutants (D374A and G443A) of the HuNoV GII.4/HS194/2009/US strain were generated in this study. The Gateway entry vector (pENTRTM/SD/D-TOPO-HS194) containing the major capsid protein VP1 gene of WT HS194.2009 strain was used as a template. The QuikChange II XL site-directed mutagenesis kit (Agilent Technology, Santa Clara, CA, USA) was used to make the site mutation in the VP1 gene in the entry vector for the mutants. To mutate the D (aspartic acid) to A (Alanine) at amino acid 374 (D374A), forward and reverse primers were: 5′-GGTACTGATACAGAAAATG*C*CTTTGAAACTCACC-3′ and 5′-GGTGAGTTTCAAAGGCATTTTCTGTATCAGTACC-3′, respectively. To mutate G (Glycine) to A (Alanine) at amino acid 443 (G443A), the forward and reverse primers were 5′-GCCCGGATGCAGCG*C*GTATCCCAACAACATGG-3′ and 5′-CCATGTTGGGATACGCGCTGCATCCGGGC-3′, respectively. After confirmation by sequencing, LR recombination was performed with the entry vector and BaculoDirect^TM^ Linear DNA to generate the recombinant baculovirus DNA carrying the mutant VP1 gene of HS194. The insect Sf9 cells were transfected by the recombination reaction products and the cells containing the recombinant baculovirus DNA were positively selected by ganciclovir. The expression of mutated HS194 capsid proteins in the Sf9 cells and culture supernatants were examined by immunoblot using the guinea pig antiserum against HS194 strain. The recombinant baculoviruses were propagated to prepare virus stocks with high titers for routine VLP expression. The production, purification and examination of GII.4.HS194 VLP mutants were performed as referenced above [16].

### 2.3. Extraction of Plant Cell Wall Material (CWM)

Extraction of lettuce leaves CWM was performed at 4 °C as described in our previous work [16]. Mature romaine lettuce heads were purchased from a local grocery store. Briefly, leaves were ground in liquid nitrogen to fine powder. The powder was then homogenized in 80% ethanol using polytron homogenizer (Cole-Parmer Instruments, Vernon Hills, IL, USA), followed by centrifugation for 20 min at 2500× *g*. The resulting pellet was subjected to a series of ethanol washes (80% twice and 100% once) and was then stirred for 2 h at 4 °C in 60 mL methanol: chloroform (1:1, *v*/*v*). The pellet was collected by filtration using Whatman^®^ glass microfiber filters grade GF/A (Sigma, St. Louis, MO, USA), washed with ice-cold methanol: chloroform and finally with ice-cold acetone. The pellet was vacuum dried overnight at RT and the resulting CWMs were stored at 4 °C in a tightly sealed bottle under dark conditions.

### 2.4. Detection of HuNoV VLP Binding to CWM Using Enzyme-Linked Immunosorbent Assay (ELISA)

The CWM-ELISA protocol was followed as previously described [16,18]. Briefly, CWMs were resuspended in PBS (pH 7.4) at 5 mg/mL, vortexed for 2 min, centrifuged twice at 500× *g* for 2 min and the resulting supernatant was used to coat MaxiSorp^TM^ 96-well plates (Thermo Fisher Scientific Inc., Rockford, IL, USA). The plates were incubated at 4 °C for 18 h. Following three washes with PBS-T, blocking was performed with 200 µL of PBS supplemented with 2% nonfat dry milk (blocking buffer) for 1 h at 37 °C. Following blocking and washing, 50 µL of HuNoV VLPs diluted in PBS (10 µg/mL) were added and the plates were incubated for 1 h at RT. For all the historical GII.4 VLPs and mutants, the primary and secondary antibodies used were rabbit anti-GII.4 VLPs and a goat anti-rabbit IgG HRP-conjugated (KPL, Gaithersburg, MD, USA) at 1:1000 and 1:2000, respectively. The rabbit anti-GII.4 norovirus polyclonal sera was generated from a rabbit hyperimmunized with HuNoV VLP cocktail including GII.4 1987, 2002, 2006b and 2009. This antibody was able to detect all GII.4 VLPs from 1987 to 2009 used in this study. This antibody was generated as described previously [33]. For the GI.1 VLPs and its mutant, the primary and secondary antibodies used were a rabbit anti-GI.1 VLP antiserum and a goat anti-rabbit IgG HRP-conjugated (KPL) at 1:3000 and 1:2000, respectively. Both antibodies were diluted in blocking buffer and incubated at 37 °C for 1 h. Plates were washed four times with PBS-T following each step. Plates were incubated with peroxidase substrate tetramethylbenzidine (TMB) (KPL, USA) for 10 min at RT. The reaction was stopped with 0.3 M sulfuric acid and the absorbance was read at 450nm. Controls included: (i) wells coated with CWM but no VLP added (named “No VLPs”); (ii) wells with CWM, VLP and secondary antibody but without primary antibody (named “No primary”); and (iii) PBS coated wells with VLP, primary and secondary antibodies added (named “No coating”).

### 2.5. Porcine Gastric Mucin and Human B-type HBGA Saliva ELISA

Purified porcine gastric mucin (PGM) type III (Sigma, USA) was used as a positive carbohydrate control since it was previously reported to contain primarily A, H and less of Le^b^ and Le^y^ antigens [16,18,28,34]. In addition, the GII.4.HS194 VLPs were shown to bind to PGM through the A and H antigens [16]. For ELISA with historical GII.4 VLPs and GI.1 VLPs, we used PGM at 1mg/mL, a concentration experimentally determined to generate an absorbance double that of HuNoV VLP binding to CWM. The ELISA protocol described above was followed.

For assessing whether the two mutations at position 374 and 443 of GII.4.HS194 affected its HBGA binding profile, binding to PGM or B-type HBGA human saliva was tested. First, to determine whether the primary antibody (rabbit anti-GII.4 VLPs) can still detect the mutant VLPs, an ELISA plate was coated with 2 µg/mL of each of the WT and mutant VLPs for 4 h at RT. Following an overnight standard blocking and washing steps, the primary antibody was serially diluted (1:10), added in duplicate to each VLP-coated wells and allowed to bind for 1 h at 37 °C while the secondary antibody (goat anti-rabbit IgG HRP-conjugated) was added at 1:2000 as described above. The primary antibody bound to WT and mutant VLPs without significant differences at all the tested dilutions; however, the binding signal for WT and mutant VLPs started to steadily decrease beyond the 1:8000 dilution (data not shown). Second, ELISA plates were coated with PGM at 5mg/mL or with a human blood type B saliva at 1:500 dilution. Briefly, 16 µg/mL of each of the two mutants and the wildtype VLPs were serially diluted (1:2), added in duplicate to the wells and allowed to bind for 1 h at 37 °C. Following standard washings, blocking and incubations as described above, the primary antibody (rabbit anti-GII.4 VLPs) was added at 1:8000, while the secondary antibody (goat anti-rabbit IgG HRP-conjugated) was added at 1:2000. The ELISA plates were stopped and read at A450nm.

### 2.6. Detection of HuNoV VLP Binding to Lettuce Tissues Using Immunofluorescence Assay (IFA)

The VLP-lettuce tissue IFA protocol was followed as previously described [16,18]. Briefly, romaine lettuce heads were purchased from a local grocery store. The leaves were cut into small pieces (0.5 × 0.5 cm^2^), dehydrated in 30 mL of gradient ethanol series (70% (*v*/*v*), 90%, 95%, 100% and 100%) for 6 h and embedded in paraffin blocks. Five-micrometer sections were cut using a microtome and collected on glass slides (Fisher Scientific, Pittsburgh, PA, USA). Slides were dried at 37 °C for 2 h, deparaffinized in xylene for 15 min and rehydrated in 30 mL gradient of ethanol series (70%, 90%, 95%, 100%, 100%, *v*/*v*). Slides were incubated with cellulase R-10 (PhytoTechnology Laboratories, Lenexa, KS, USA) at a final concentration of 1 µg/µL at 37°C for 1 h. Following washing three times with 300 mL of PBS containing 0.05% Tween-20 (PBS-T), blocking was performed with 200 µL of PBS supplemented with 1% bovine serum albumin (BSA). For fucosidase digestion, each slide was digested with 20 U α-1,2 fucosidase (NEB, Ipswich, MA, USA) at 37 °C for 1 h. For inhibition with HBGA monoclonal antibody or lectins, a 200 µL of anti-H type 1 HBGA monoclonal antibody BG4 (Covance, Emeryville, CA, USA) diluted in PBS were added to the slides and incubated for 1 h at 37 °C. Plant lectins DBA (*Dolichos biflorus*), LEA (*Lycopersicon esculentum*), BS-I (*Bandeiraea simplicifolia*) and LcH (*Lens culinaris*), which recognize GalNAc (type A HBGA), GlcNAc, α-D-Gal (type B HBGA) and α-D-Man/α-D-Glc, respectively, were diluted in TBS buffer (50 mM Tris-HCl, 150 mM NaCl, supplemented with 2.5 mM MnCl_2_ and CaCl_2_) to a final concentration of 100 µg/mL added to the slides and incubated for 1 h at 37 °C. Subsequently, the slides were incubated with 200 µL of HuNoV VLPs (final concentration of 10 µg/mL) at RT for 1 h. The bound VLPs on lettuce tissues were detected using guinea pig anti-GII.4 NoV VLPs or rabbit anti-GI.1 VLP antisera (1:1000) and Alexa Fluor 488-conjugated goat anti-guinea pig or anti-rabbit IgG (Invitrogen) at 1:700 dilution (green signal). Negative controls used included slides treated as above but without the VLPs. The slides were washed three times with 300 mL PBS-T after each incubation step. The plant chloroplast autofluorescence was shown in red while nuclei were stained with 4’,6-diamidino-2- phenylindole (DAPI) and shown in blue. All pictures were taken using the same exposure time.

### 2.7. Statistical Analyses

GraphPad Prism^®^ version 5 (GraphPad Software, LaJolla, CA, USA) was used for statistical analyses. One-Way ANOVA followed by Tukey’s post hoc test was used to determine significant differences between the different treatments. Each treatment in each ELISA assay was run in triplicate and the ELISA was repeated at least twice to determine assay consistency. The VLP mutants (D37A and G443A) dose response ELISA with B-type saliva and PGM were only performed once with duplicate wells but with multiple VLP concentrations. The Alexa Fluor 488 green fluorescent intensity of all IFA images was measured by ImageJ software (https://imagej.nih.gov/ij/). The mean fluorescent intensity for WT VLPs (GII.4 or GI.1) was transformed to 100% and the intensity values of the different treatments were adjusted accordingly. Differences in means were considered significant when the *p*-value was <0.05. Data were represented as mean ± standard errors. Significantly different means were denoted by different alphabetical letters. The binding of VLPs to PGM or lettuce CWM was considered positive when the A_450_ signal was significantly higher than the highest A_450_ of control negative wells.

## 3. Results

### 3.1. Binding of Historical GII.4 VLPs to Lettuce CWM Correlated with Their PGM Binding

**Figure 1:**
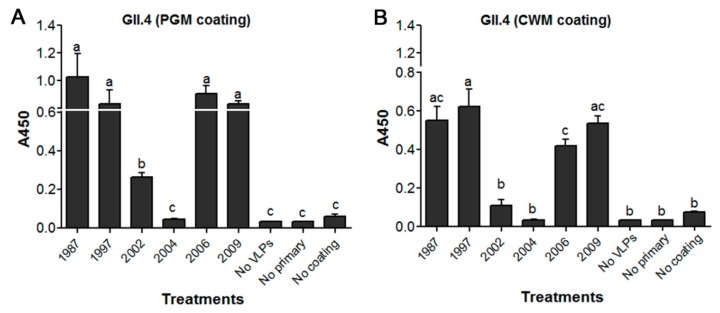
Enzyme linked immunosorbent assay (ELISA) screening of historical HuNoV GII.4 virus-like particles (VLPs) for their binding to (**A**) Porcine gastric mucin (PGM) and (**B**) lettuce leaves cell wall material (CWM). Means with different letters differ significantly.

The binding of our previously characterized strain GII.4/HS194/2009 to PGM was not significantly different from that of the GII.4 historical strains of 1987, 1997 and 2006 (Figure 1A). The GII.4.2002 VLPs showed significantly reduced signal in comparison to the 1987, 1997, 2006 and 2009 VLPs (Figure 1A). However, the binding of GII.4.2004 VLPs to PGM was not statistically different from negative control wells (Figure 1A). In a similar trend to PGM, the GII.4 of 1987, 1997, 2006 and 2009 exhibited a higher binding signal to lettuce CWM than that of GII.4 of 2002 and 2004 (Figure 1B). The signal of GII.4.2002 and GII.4.2004 was not significantly different than that of the negative control wells (Figure 1B).

### 3.2. Loss of HBGA Binding Profile for GII.4 D374A and G443A VLP Mutants

**Figure 2:**
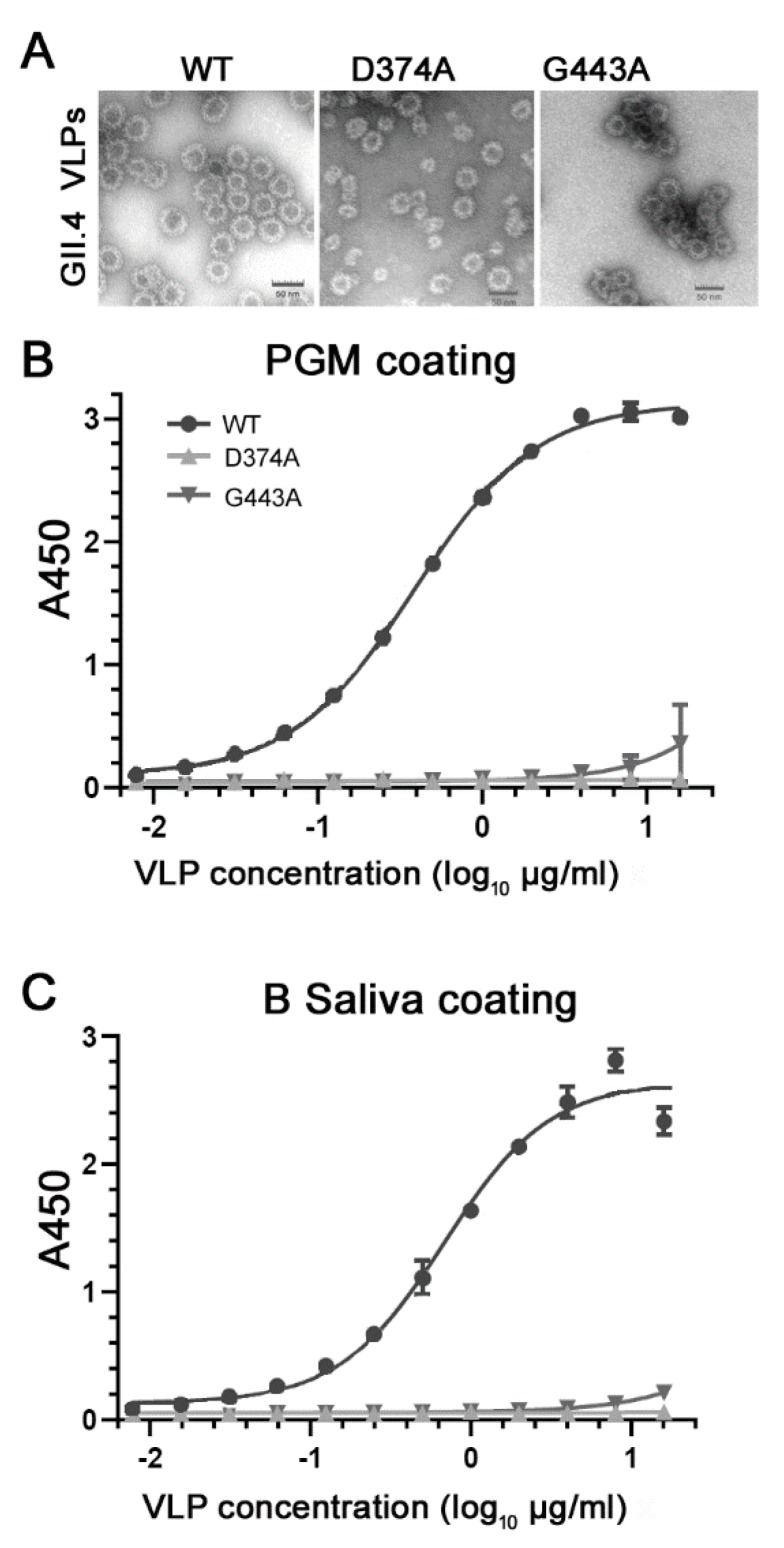
Characterization of the VLPs of HuNoV GII.4.HS194.2009 WT and its two mutants D374A and G443A by (**A**) transmission electron microscopy to show their sizes and morphology and by (**B**) PGM ELISA and (**C**) human B-type HBGA saliva ELISA to detect their binding to HBGA.

The VLPs of the two mutants D374A and G443A showed typical morphology and size (~40 nm in diameter) under TEM as those of the GII.4.HS194.2009 WT VLP (Figure 2A). Besides the 40 nm particles, the VLPs of the mutant D374A also formed particles with a 27 nm in diameter (Figure 2A). Neither VLP mutant showed binding to PGM (Figure 2B) or the B-type HBGA human saliva (Figure 2C) at all concentrations tested, with the exception of some binding of mutant G443A at high concentrations 10–16 µg/mL with *A*_450_ values of ≤ 0.4. In contrast, the WT VLPs binding to PGM (Figure 2B) or the B-type HBGA human saliva (Figure 2C) increased with increasing VLP concentrations.

### 3.3. Amino Acid Residues 374 and 443 are Essential for GII.4 Binding to Lettuce

**Figure 3:**
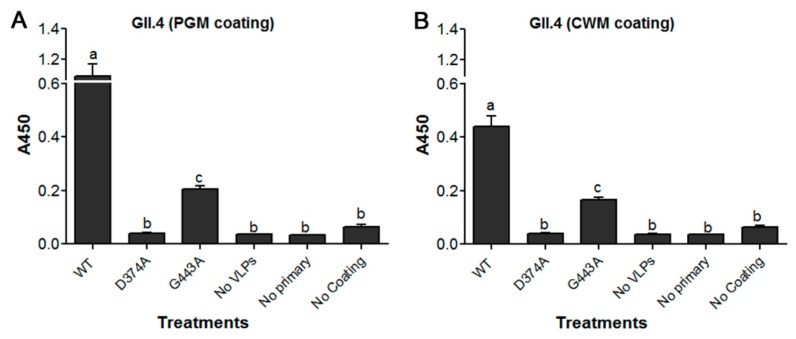
ELISA showing the binding of HuNoV GII.4 WT VLPs and its two mutants D374A and G443A VLPs to (**A**) PGM and to (**B**) lettuce CWM. Means with different letters differ significantly.

**Figure 4:**
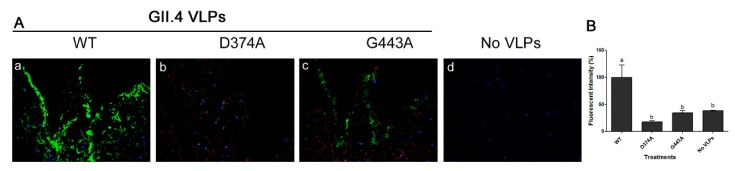
(**A**) Immunofluorescence assay (IFA) showing the binding of (a) GII.4 WT VLPs and its two mutants (b) D374A and (c) G443A VLPs to lettuce tissues as well as (d) control negative with no VLP added, Magnification: 40×. (**B**) Quantification of fluorescent intensity. Means with different letters differ significantly.

Mutation of WT GII.4.HS194.2009 at amino acid residue 374, abolished VLP binding to PGM, as its binding signal was similar to that of negative control wells (Figure 3A). However, mutation at the amino acid residue 443 significantly reduced the VLP binding to PGM by over 80%. The binding of mutant G443A VLPs to PGM was significantly higher than negative control wells (Figure 3A). Binding of the two mutant VLPs to lettuce CWM showed a similar trend as their binding to PGM (Figure 3B). Specifically, mutation of the WT at residue 374 abolished its binding to lettuce CWM, while mutation at residue 443 significantly reduced VLP binding by over 60%. Using IFA, the WT GII.4.HS194.2009 VLPs were shown to bind to lettuce tissues (Figure 4Aa), while slides with no VLP added had no signal (Figure 4Ad). The VLP mutant D374A lost its binding to lettuce tissues (Figure 4b), while mutant G443A showed weak IFA binding signal (Figure 4Ac). Specifically, the fluorescent intensity of GII.4 WT was significantly different from that of D374A and G443A mutant VLPs and the negative control. Mutant D374A showed approximately 82% loss of fluorescent signal as compared to WT VLPs, while G443A showed a 65% loss in fluorescent signal (Figure 4B).

### 3.4. HuNoV GI.1 Binds to Lettuce Tissues Through a Fucose Moiety on H-like HBGA

**Figure 5:**
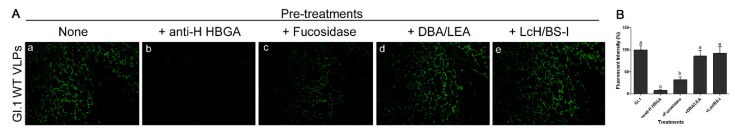
(**A**) Immunofluorescence assay showing the binding of (a) GI.1 VLPs to the lettuce tissues without any pre-treatment and following pre-treatments with (b) anti-H HBGA antibody, (c) fucosidase enzyme or lectins (d) DBA/LEA and (e) LcH/BS-1, Magnification: 40×. (**B**) Quantification of fluorescent intensity. Means with different letters differ significantly.

The binding of GI.1 to lettuce was visualized using IFA (Figure 5Aa). Pre-treatment of the lettuce slides with monoclonal antibody against H-type HBGA eliminated the VLP IFA signal (Figure 5Ab), while pre-treatment with fucosidase highly reduced the signal (Figure 5Ac). In contrast, a pre-treatment with lectins DBA (recognizing A-type HBGA) and LEA (recognizing GlcNAc) or LcH (recognizing α-D-Man/α-D-Glc) and BS-I (B-type HBGA) had no effect on the GI.1 VLP IFA signal (Figure 5Ad,e). Specifically, the fluorescent intensity of GI.1 binding to lettuce tissues was only significantly different from that of anti-H HBGA and fucosidase pre-treatments but not from those of lectin pretreatments. Pre-treatment with anti-H HBGA and fucosidase reduced the fluorescent signal by approximately 92 and 67% as compared to that of GI.1 signal (Figure 5B).

### 3.5. Amino Acid at Position 375 is Important for GI.1 Binding to Lettuce

**Figure 6:**
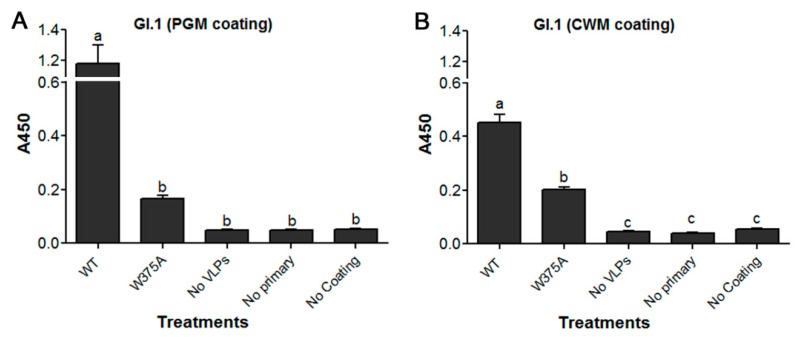
ELISA showing the binding of HuNoV GI.1 WT VLPs and its mutant W375A VLPs to (**A**) PGM and (**B**) lettuce CWM. Means with different letters differ significantly.

**Figure 7:**
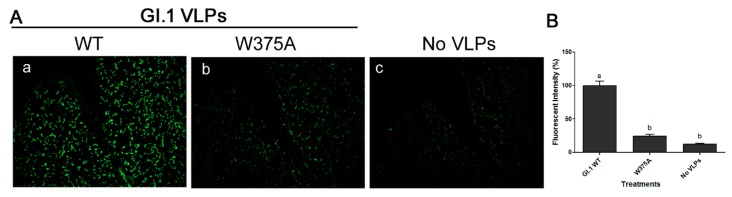
(**A**) Immunofluorescence assay showing the binding of (a) GI.1 WT VLPs and (b) its mutant W375A VLPs to lettuce tissue, as well as (c) control negative with no VLP added, Magnification: 40×. (**B**) Quantification of fluorescent intensity. Means with different letters differ significantly.

HuNoV GI.1 VLPs bound to PGM, while its mutant W375A exhibited significantly reduced binding (Figure 6A). The binding of the mutant VLPs was reduced by ~85% and was not significantly different from the three negative controls. Binding of GI.1 and its mutant VLPs to lettuce CWM showed a similar trend to their binding to PGM (Figure 6B). Specifically, GI.1 VLPs bound to lettuce while its mutant W375A VLPs, exhibited significantly reduced binding by ~55%. The binding of the mutant W375A was significantly higher than any of the three negative controls (Figure 6B). Mutant W375A showed a weak IFA signal, which is nearly comparable to the negative control slide (Figure 7Ab,c). Specifically, the fluorescent intensity of G1.1 WT VLP was significantly different than that of W375A mutant VLPs and the negative control. Mutant W375A showed approximately 75% loss of fluorescent signal as compared to WT VLPs (Figure 7B).

## 4. Discussion

Our research group was the first to establish the involvement of carbohydrates in the specific binding of HuNoV GII.4 to lettuce [16]. We also were the first to discover that this binding is mediated through a fucose moiety in H-like HBGA, which is present in the cell wall hemicelluloses of lettuce leaves [18]. Because the virus is known to bind human HBGA through a number of amino acid residues present on the P2 subdomain of the major capsid protein (the HBGA binding sites), our hypothesis was that HuNoV binding to lettuce carbohydrates is mediated through the virus HBS. Our previous work was based on HuNoV strain GII.4.HS194.2009, which is a member of the Minerva/2006 subtype of the predominant genotype globally. For this reason, we first screened the binding potential of a number of GII.4 historical strains which accumulated natural mutations throughout the years that resulted in changes to their HBGA binding profiles (Table 1) [21]. The premises was that if the binding to lettuce CWM is mediated through the virus HBS, then these natural mutants of GII.4 should bind to lettuce in a manner reflective of their HBGA binding profiles. Indeed, our results showed that historical GII.4 VLPs bound to lettuce CWM in a similar profile to their binding to PGM (used as a proxy for binding to HBGAs), indicating that the binding is dependent on the HBGA phenotype of each strain, that is, mediated through the virus HBS. Specifically, because the GII.4.2002 was shown to bind to Le^y^ antigen and less efficiently to H3 (Table 1), it was expected that it would bind less efficiently to PGM (which primarily has A and H antigens). Likewise, GII.4.2002 did not bind to lettuce leaves which primarily have H-like HBGA antigen [18]. The GII.2004 strain provided additional evidence that HuNoV binding to lettuce is mediated through the HBS. The VLPs of GII.4.2004 strain used in this study were shown not to bind to any known synthetic or saliva HBGA carbohydrates [21,24] and this is in agreement with our PGM-ELISA data which predicted the “no binding” result obtained with lettuce leaf CWM. There have been conflicting reports regarding the 2004 VLP variants, as two other groups using the P particles (expressed from the P domain) and VLP binding by surface plasmon resonance reported that strains from the GII.4.2004 bind to various HBGAs. This could be due to the different GII.2004 virus isolates (TCH05 and E1057) originally used in the preparation of the P particles or VLPs [25,35] versus the one used in our study which was commercially synthesized (Hunter284E.04O.AU) [29] and may not be 100% representative of the Hunter cluster. Regardless, the GII.4.2004 strain that we used in this study did not bind to any synthetic HBGA [21,24] and thus served along with GII.4.2002 VLPs as strains lacking binding to HBGA or efficient binding to the H antigen, respectively. The remaining historical GII.4 of 1987, 1997 and 2006 are known to bind to various HBGAs including the H antigen and therefore they were expected to show binding to PGM. Their binding to lettuce CWM in a manner reflective of their binding to PGM, provided further evidence in support of our hypothesis. Taken together, our results suggest that GII.4 HuNoVs binding to lettuce is mediated through the virus HBS and is dependent on their efficient binding to H-type HBGA.

Multiple sequence alignment of representative GII.4 strains from 1995 to 2010 revealed that certain amino acids are conserved (residues 342–347, 374 and 440–444—named site 1), while others (residues 389–395—named site 2) are temporally evolving [25]. Binding of GII.4 to the fucose moiety of H was shown to be mediated through site 1 while that of Lewis antigens was shown to be mediated through both site 1 and site 2 [25]. In addition, the variability of amino acids in site 2 was suggested to modulate the affinity for a particular Lewis HBGA or particular combination of HBGAs. Therefore, we selected two amino acids (at positions 374 and 443 of site 1) for mutagenesis, which are located within site 1 and are thus common among GII.4 strains. Two crystallography studies indicated that these two amino acids in GII.4 HuNoVs bind the α-fucose of A and B trisaccharides [36] or that of H-type pentasaccharide [25]. Because we have shown that HuNoV GII.4.HS194 VLPs interacts through a fucose moiety in the lettuce H-like HBGA, it is therefore expected that mutating these residues would significantly reduce the binding to lettuce. As expected, our results showed that mutating residue 374 completely abolished the binding to PGM and to lettuce CWM, while that of 443 showed significantly reduced binding to both PGM and lettuce. This could be due to the different ways in which these two amino acids interact with the fucose moiety of the HBGA. Crystallographic analysis showed that both the side chain carboxyl and oxygen groups of aspartic acid residue at 374 form stable and strong hydrogen bonds with the hydroxyl groups of the fucose ring [36]. In contrast, there is a potential hydrogen bond between the backbone amide group of Glycine residue at 443 and the O atom of the fucose ring. Consistent with our PGM results, a mutation in residue 374 (D374A) of a GII.9 strain completely abolished its binding to H and Lewis antigens as confirmed by a saliva-based binding assay [37]. Similarly, a previous study in which a mutation at 443 (G443A) was performed in a GII.4 strain, showed that binding of VLP mutant G443A to HBGA glycoconjugates was absent or minimal as assayed by ELISA on synthetic carbohydrates linked to bovine or human serum albumin [35]. The minimal binding observed in our study for this VLP mutant (G443A) could be the result of using more biologically relevant HBGAs found in PGM which is known to have higher affinity to the virus than synthetic saccharides [36]. Taken together, our results showed that amino acid residues such as those at positions 374 and 443 located in the HBGA binding sites are important for HuNoV GII.4 binding to lettuce, thus providing additional evidence supporting our hypothesis.

Although HuNoV GII.4 is the predominant strain globally and is often implicated in leafy green outbreaks [38,39,40,41], the GI.1 is also implicated in HuNoV foodborne outbreaks [4]. The GI.1 HuNoV binds to similar HBGAs (A and H antigens) as the GII.4.HS194.2009 strain that we previously assessed (Table 1); however, they differ considerably in their amino acid sequence at the HBS [22]. Interestingly, our results indicate that GI.1 binds to lettuce in a similar manner to GII.4.HS194.2009, that is, through a fucose moiety on an H-like HBGA and not through A or B antigen. The latter is supported by our previous results showing that lettuce tissues contain the H-like HBGA antigen but not the A- or B-type [18]. To determine whether GI.1 binding to lettuce CWM was mediated through its HBS, we utilized the previously characterized GI.1 VLP mutant (W375A) that was shown by surface plasmon resonance assay, to lose binding to both A and H-type HBGAs [22]. Our results showed that this mutant showed minimal binding to PGM that was not statistically different from that of the negative wells. However, it still bound to lettuce tissues, although the binding was significantly reduced by over half of that of the WT. This result may be explained by examining the complex interaction resolved through crystallography for GI.1 interacting with H-type HBGA pentasaccharide [22]. The latter study showed that although the tryptophan residue at 375 is conserved among the GI HuNoV, it does not provide hydrogen bonding to the fucose of the H-antigen. Rather, hydrophobic interactions are involved between Trp-375 and the α-Fuc of the H-type HBGA and this interaction is conserved and plays a critical role in conferring carbohydrate specificity [22]. In addition, synthetic carbohydrates such as the H pentasaccharride used in the crystallography or the H trisaccharide used in the surface plasmon resonance detection assays [22] may not completely represent the actual biologic H antigen present in PGM or that of H-like HBGA present in lettuce used in our study. This issue is a known limitation to many structural studies that utilize smaller saccharrides (such as tri or penta) and the P domain of the HuNoV. Natural HBGA have higher affinities to the virus than that of the P particles to the synthetic trisaccharides and this has been used to suggest that additional interactions must occur between the virus and natural HBGA beyond those identified in crystallography studies [36]. Therefore, mutant W375A may still interact with lettuce carbohydrates through other weaker interactions. Regardless, overall our results suggest that GI.1 interaction with lettuce is similar to that of GII.4 and is mediated in part through the virus HBGA binding sites, thus also supporting our initial hypothesis.

## 5. Conclusions

In conclusion, we have shown that the binding of HuNoV GII.4 and GI strains to lettuce is mediated through the virus HBGA binding sites. HuNoV GI.1 was shown to bind to lettuce in a similar fashion to GII.4, that is, through H-like HBGA antigen. The binding of historical HuNoV strains to lettuce correlated with their ability to bind to HBGAs. The lettuce binding profiles of historical GII.4 HuNoV strains which mimicked their PGM (i.e., HBGA) binding profiles as well as the mutants of GI.1 and GII.4 which lost HBGA binding resulting in loss or significant reduction in binding to lettuce provided multiple evidence to support our hypothesis. Overall, our research suggests that integrating epidemiological investigation during norovirus foodborne outbreaks with genotyping data will allow for a better assessment of the relationship between circulating/emerging norovirus genotypes and lettuce and the impact on foodborne disease.

## Figures and Tables

**Table 1:** A list of virus-like particles of GI.1 and GII.4 used in our study and their histo blood group antigen (HBGA) binding profiles as adapted from previous studies [21,22,24,28,29]. HBGA profile for GII.4.HS194 was generated in this study as described previously [30,31].

Genotype	Year	Strain	GenBank Accession No.	VLP Variant	VLP Name	HBGA Profile
GI.1	1968	Norwalk	M87661	Hu/US/1968/GI.1/Norwalk	GI.1.1968	H1, H3 and A
GII.4	1987	Camberwell	AAK50355.1	Hu/GII.4/MD145-12/1987/US	GII.4.1987	H3 and Le^y^
	1997	US95/96	AFJ04707.1	Hu/GII.4/1997/USA	GII.4.1997	H3, A, B, Le^y^ and Le^b^
	2002	Farmington Hill	AFJ04708.1	Hu/GII.4/Farmington Hills/2002/USA	GII.4.2002	Le^y^, H3 and B
	2004	Hunter	AAZ31376.2	Hu/GII.4/Hunter 284E/2004/AU	GII.4.2004	None
	2006	Minerva	AFJ04709.1	Hu/GII.4/Minerva/2006/USA	GII.4.2006	H3, B and A
	2009	HS194	GU325839	Hu/GII.4/Minerva/2006/US	GII.4.HS194	H3, B, H1, Le^y^, A and Le^b^
